# Coating of intravascular balloon with paclitaxel prevents constrictive remodeling of the dilated porcine femoral artery due to inhibition of intimal and media fibrosis

**DOI:** 10.1007/s10856-016-5737-y

**Published:** 2016-07-07

**Authors:** Noemi Pavo, Eslam Samaha, Inna Sabdyusheva, Rembert Pogge von Strandmann, Stefanie Stahnke, Christian A. Plass, Katrin Zlabinger, Dominika Lukovic, Zoltan Jambrik, Imre J. Pavo, Jutta Bergler-Klein, William A. Gray, Gerald Maurer, Mariann Gyöngyösi

**Affiliations:** 1 Department of Cardiology, Medical University of Vienna, Währinger Gürtel 18-20, Vienna, 1090 Austria; 2 Department of Pulmonology, Medical University of Vienna, Vienna, Austria; 3 Eurocor GmbH, Bonn, Germany; 4 Heart Center, Semmelweis University, Budapest, Hungary; 5 Center for Interventional Vascular Therapy, Columbia-Presbyterian Hospital, New York, NY USA

## Abstract

Here we investigated how a coating of intravascular balloon with paclitaxel (drug-coated balloon; DCB, Freeway™) impacted porcine peripheral artery vascular function and remodeling. Domestic swine (n = 54) underwent percutaneous overstretch balloon dilation of femoral and iliac arteries, controlled by angiography and optical coherence tomography (OCT). Paclitaxel tissue uptake was measured at 1 h and 1, 3, and 9 days post-dilation. At these time-points and at 32 ± 2 days, vascular function of the dilated arteries was assessed using the organ chamber model. Neointimal growth and remodeling indices were determined using OCT and histology at 32 ± 2 days. Intima and media fibrosis were quantified by picrosirius red staining. Post-inflation femoral artery tissue drug levels were 460 ± 214, 136 ± 123, 14 ± 6, and 0.1 ± 0.1 ng/mg at 1 h and 1, 3, and 9 days, respectively. Compared to plain balloon, Freeway™ resulted in a significantly smaller neointimal area (*P* < 0.05), less tunica intima (8.0 ± 5.4 vs 14.2 ± 4.7 %) and media fibrosis (15.6 ± 7.7 vs 24.5 ± 5.4 %), and less femoral artery constrictive remodeling (remodeling index: 1.08 ± 0.08 vs 0.94 ± 0.08). The DCB was associated with significantly increased vasoconstrictor tone and endothelium-dependent vasodilation impairment shortly after post-overstretch injury. Overall, DCB dilation of peripheral arteries resulted in high drug uptake into arterial tissue. Compared with the plain balloon, the DCB was associated with decreased vessel wall fibrosis after balloon overstretch injury, and reduced degrees of constrictive remodeling and neointimal hyperplasia.

**Electronic supplementary material:**

The online version of this article (doi:10.1007/s10856-016-5737-y) contains supplementary material, which is available to authorized users.

## Introduction

As treatment for atherosclerotic peripheral artery disease, percutaneous transluminal angioplasty shows limited success due to high restenosis rates [1–3], with 1-year patency rates of 75–85 % after bare-metal stent (BMS) placement [4–6]. Studies testing the implantation of drug-eluting stents (DES) in the superficial femoral artery to prevent restenosis have shown conflicting results, with DES showing advantages over BMS in the short term (6 months) [3], but with diminishing benefits over a longer time [6]. Sirolimus-coated stents are more consistently superior for treating focal infrapopliteal lesions when compared with BMS [7]. In the Zilver PTX randomized study of patients with peripheral artery disease, implantation of polymer-free DES led to improved 12-month results compared to balloon dilation, with similar superiority results when balloon dilation was followed by provisional DES stenting [8].

The use of drug-coated balloons (DCBs) represents an alternative approach to DES for local antiproliferative drug delivery into the vessel wall [9–13]. DCB employment in peripheral endovascular procedures has been successfully demonstrated in pre-clinical [14–17] and clinical [18–24] studies.

Optical coherence tomography (OCT) has recently been introduced into clinical practice to allow in vivo visualization of stent strut coverage with neo-endothelium [25]. Correct imaging of femoral or iliac vessels is technically challenging due to the large size of the peripheral arteries. Accordingly, current publications describing OCT use in peripheral arteries are limited to case reports [26, 27] and ex vivo imaging [28].

We previously demonstrated that drug deposition in coronary arteries leads to time-dependent impairment of vascular function, despite decreased restenosis [29]. Unlike in coronary arteries, the long atherosclerotic lesions of human peripheral vessels can exhibit both adaptive and constrictive remodeling within a single vessel [30], carrying potential consequences of both vessel thrombotic occlusion and severe restenosis. No systematic investigation has yet reported how peripheral arterial wall remodeling is impacted by percutaneous intervention with a paclitaxel-coated balloon, and any related mechanical injury.

The present pre-clinical investigation aimed to assess the vascular response to antiproliferative drug accumulation in the peripheral arteries after balloon dilation with the paclitaxel-coated balloon Freeway™ (Eurocor, Germany). The impact was examined in terms of vessel remodeling and vasodilatory or vasoconstrictive capacity of the femoral or iliac arteries compared to with plain balloon dilation.

## Materials and methods

### Local drug-delivery device

The Freeway™ balloon is a peripheral dilation balloon for use in humans. Its surface is coated with 3.0 µg/mm^2^ paclitaxel using the shellac-coating technique. The balloon’s tri-fold design protects the drug coating from early wash-off into the peripheral circulation while the balloon is introduced.

### Experimental design

This study was approved by the Ethical Committee on Animal Experiments at the University of Kaposvar, Hungary. Our study protocol conformed to the “Position of the American Heart Association on Research Animal Use,” adopted by the AHA on November 11, 1984.

**Fig. 1:**
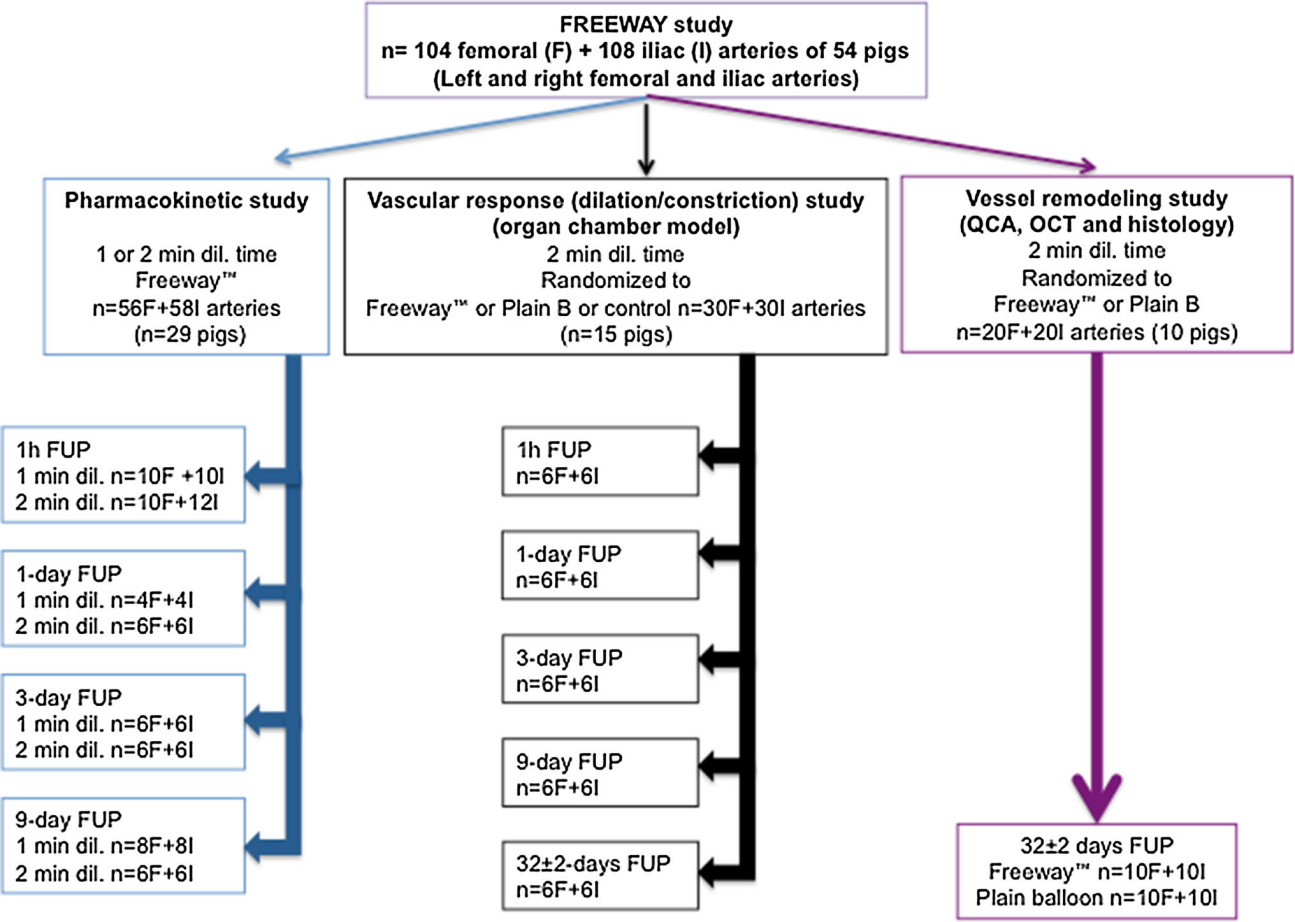
Study design, including pharmacokinetic, vascular response (physiological), and vessel remodeling (with additional safety and efficacy) investigations

The design of this prospective study is illustrated in Fig. 1, and described in detail in the Supplementary Materials and methods. Briefly, domestic pigs (weight 30–40 kg, n = 54) were pre-treated with 300 mg clopidogrel and 250 mg acetylsalicylic acid. After overnight fasting, the pigs were anesthetized for the peripheral intervention. Into the right and left femoral and iliac arteries (four vessels in each pig), we introduced the Freeway™ or plain balloon (femoral: diameter of 5.0 or 6.0 mm and length of 20 mm; iliac: diameter of 7.0 or 8.0 mm and length of 40 mm). The balloons were then inflated with 6–12 atm pressure to achieve a 1.3:1 balloon/artery ratio, producing overstretch injury. Balloon inflation was controlled by angiography, and the inflated balloon size was related to the vessel size displayed by the baseline angiography. The duration of balloon inflation was 60 or 120 s, depending on the experimental setup. The animals were allowed to recover after the procedure. Upon completing the predetermined follow-up time, the animals were humanely euthanized with saturated potassium chloride.

#### Pharmacokinetic study

Blood samples were taken at 5, 10, 20, and 60 min after the interventional procedure. At 1 h and 1, 3, and 9 days of follow-up, the paclitaxel concentration in femoral and iliac arterial tissue was measured using high-performance liquid chromatography (AnaKat Institut für Biotechnologie GmbH, Berlin, Germany) [31, 32]. In five animals, the proximal and distal reference segments of five iliac and femoral arteries (2-min dilation groups) were dissected. All balloon remnants were stored for measurements of paclitaxel on the surface.

#### Vascular response study measuring vasoconstrictor tone and endothelium-dependent and -independent vasodilation

Vasomotor reactions—including contraction, and endothelium-dependent and -independent vasodilation—were measured using the organ chamber model, as previously described [29] (Supplementary Materials and Methods).

#### Vascular remodeling study

Our investigation of vascular remodeling included OCT and histology analyses. During a follow-up period of 32 ± 2 days, the pigs received a daily dose of 75 mg clopidogrel and 100 mg acetylsalicylic acid. At the end of the study, angiography and OCT imaging were performed, and then the animals were euthanized. For histopathological and histomorphometric analyses, the peripheral arteries were flushed with 100 mL saline, followed by pressure fixation in 4 % buffered formaldehyde at 100–110 mmHg for 30 min.

### OCT imaging and analysis

For OCT imaging, a Dragonfly™ catheter (St. Jude Medical, Lightlab Imaging, Inc., Westford, MA) was placed 3 cm distal to the affected peripheral artery segment, and images were acquired via motorized pullback [25]. The images were digitally stored using the ILUMIEN System (St. Jude Medical, St. Paul, MN). The large artery size prohibited blood clearance with a single contrast medium flush. Thus, OCT image acquisition was combined with proximal gentle balloon occlusion of the artery using low balloon inflation pressure [26]. The analysis is described in detail in the Supplementary Materials and Methods. Briefly, for the lesion with the highest degree of stenosis, we determined the remodeling index as the IEM area of the lesion divided by the IEM area of the reference segment (mean IEM of the proximal and distal reference segments within 1 cm of the lesion edge) [25]. A remodeling index of >1 or <1 was considered to indicate adaptive or constrictive remodeling, respectively.

### Histopathology and histomorphometry

The formalin-fixed dilated segments were cut into distal, mid, and proximal segments. These pieces were embedded in paraffin, cut into 4– 6-µm-thick slices, and routinely stained with hematoxylin–eosin and modified Movat’s pentachrome and picrosirius staining. We used planimetry to quantify fibrosis of the tunica intima, media, and adventitia as described elsewhere [33]. All histopathological, histomorphometric, and immunohistochemical analyses were performed in accordance with published guidelines by experienced investigators who were blinded to the treatment (Supplementary Materials and Methods). The remodeling index was calculated as the external elastic lamina (EEL) area of the lesion with the highest grade of stenosis divided by the EEL area of the reference segment (mean value of the proximal and distal reference EEL area).

### Statistics

The continuous parameters of the angiographic, histologic, and physiologic analyses are expressed as mean ± standard deviation. Tissue and plasma paclitaxel levels are presented as mean ± standard error. The continuous variables of the plain balloon and Freeway™ groups were compared using the two-sided Student’s *t* test with a significance level of 0.05. All measurements were performed offline by an independent observer who was blinded to the randomization group. For all OCT images, the lumen area of the selected region of interest was repeatedly assessed by one observer, as well as assessed by a second observer, enabling calculation of the intra- and inter-observer variability of the OCT measurements. Statistical analyses were performed using SPSS for Macintosh version 19.

## Results

**Fig. 2:**
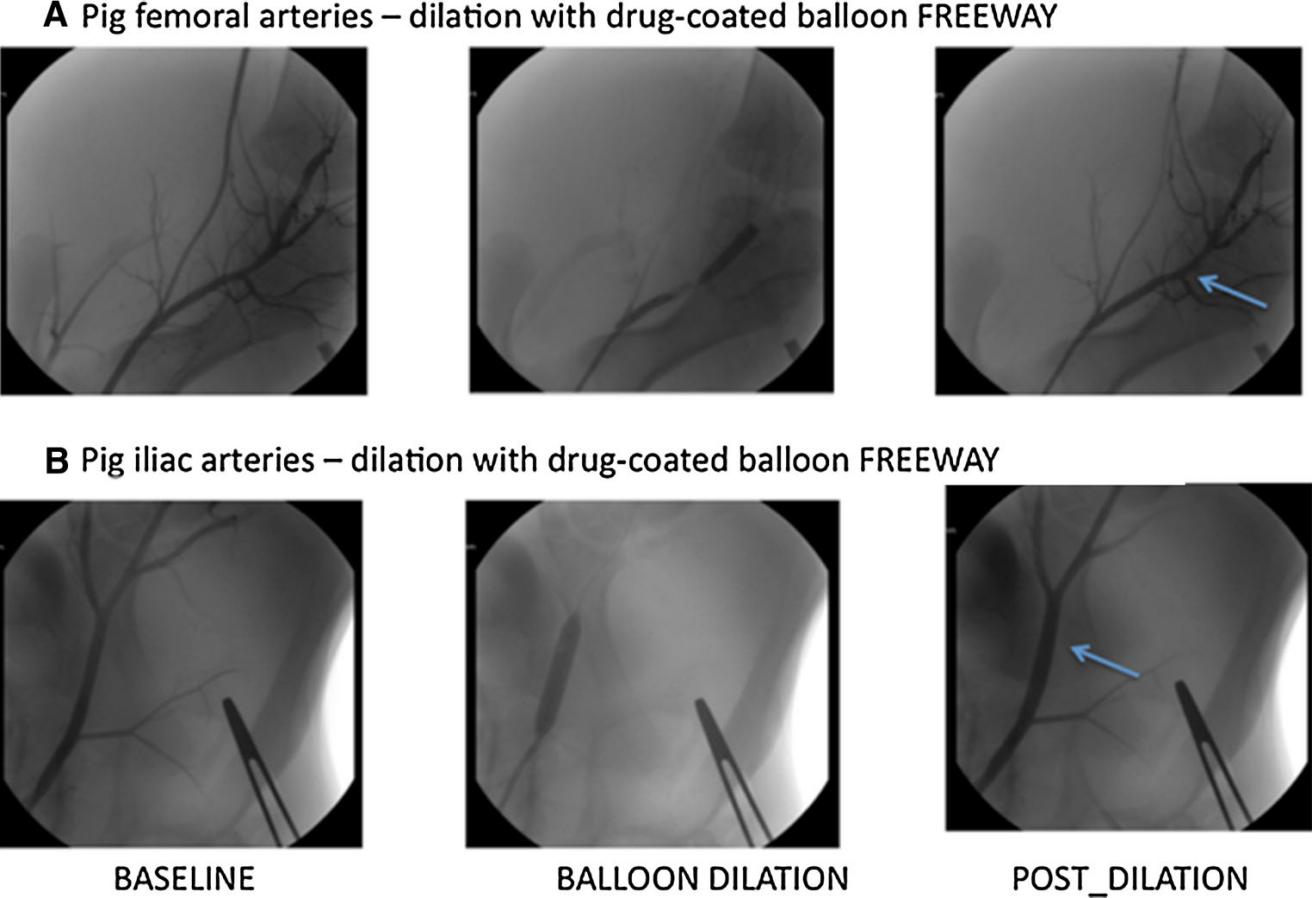
Angiography of the femoral and iliac arteries before, during, and after balloon dilation. **a** Porcine superficial femoral artery at baseline, during balloon dilation, and post-dilation. Severe post-dilation overstretch injury of the artery, including vessel perforation and perivascular contrast medium deposition (*arrow*). **b** Porcine arteria iliaca externa artery at baseline, during balloon dilation, and post-dilation. Acute vessel dilation due to injury of the adventitia following overstretch injury *(arrow)*

Figure 2 depicts the baseline anatomy of the porcine superficial femoral and iliac arteries. The common iliac artery was longer and wider than the femoral arteries, having a size similar to human femoral arteries and thus being better suited for testing human DCBs. The iliac arteries are more elastic, with collagen and elastin fibers dominating in the tunica media, and relatively few smooth muscle fibers. In contrast, the femoral arteries are muscular-type arteries, showing predominant content of smooth muscle cells.

**Fig. 3:**
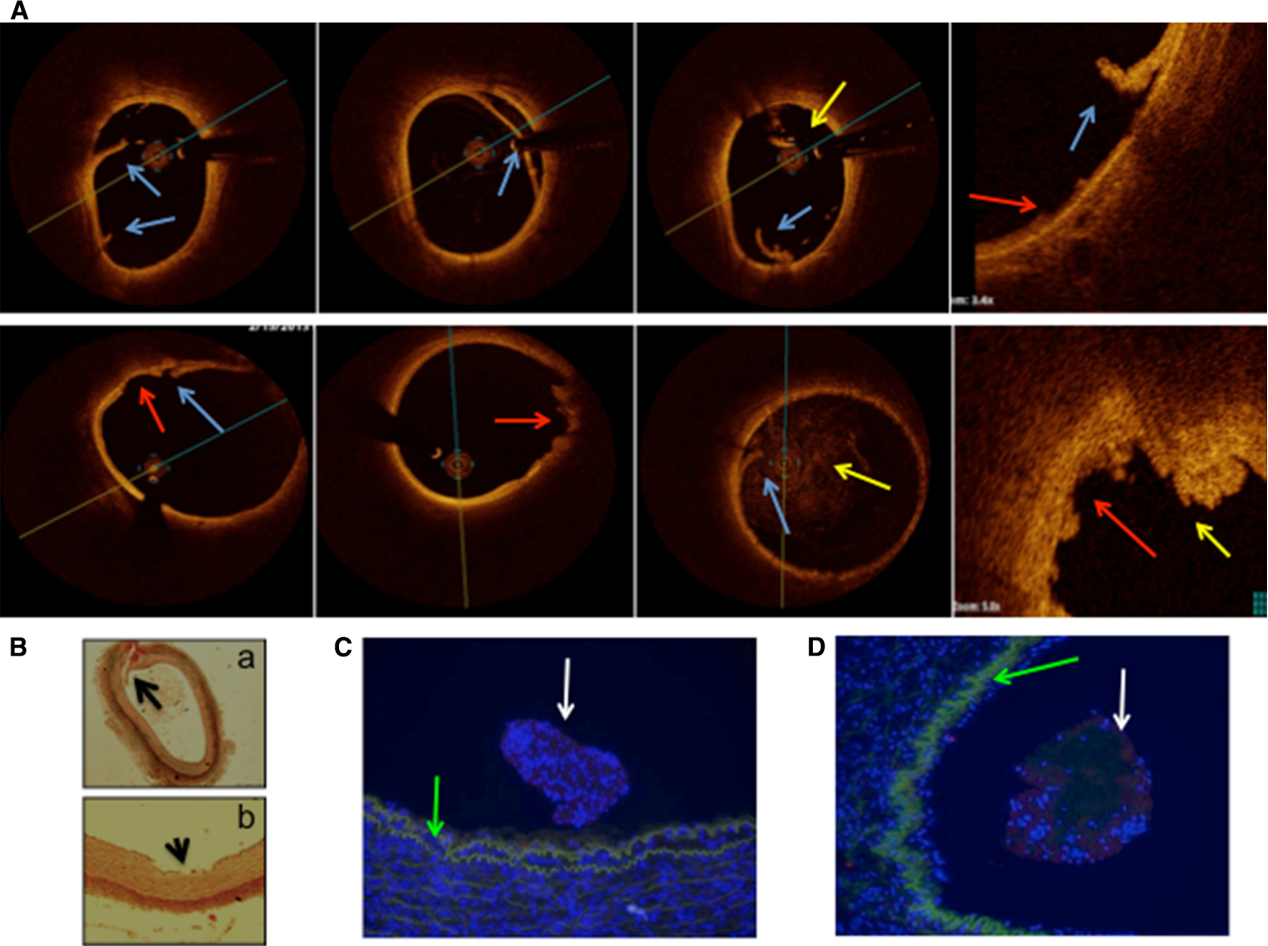
OCT and histologic imaging of balloon overstretch injury of the peripheral (femoral and iliac) arteries. **A** OCT imaging, showing intima and media rupture (*red arrow*), vessel dissection (*blue arrow*), and platelet-rich white thrombus (*yellow arrow*). **B** Histology, showing (**a**) artery rupture with hemorrhage (*black arrow*) and (**b**) severe intima and media injury (*black arrow*). Swine iliac arteries at 1 h after balloon overstretch injury, shown with MOVAT staining and under ×4(a) and ×8(b) magnification. **C, D** Tumor necrosis factor (TNF)-alpha immunofluorescence staining. Cell-rich (**c**) and cell-poor (**d**) intraluminal thrombus, with TNF-alpha expression of the platelets and inflammatory cells (*red color*). Autofluorescence of the endothelium (*green arrow)* (Color figure online)

**Fig. 4:**
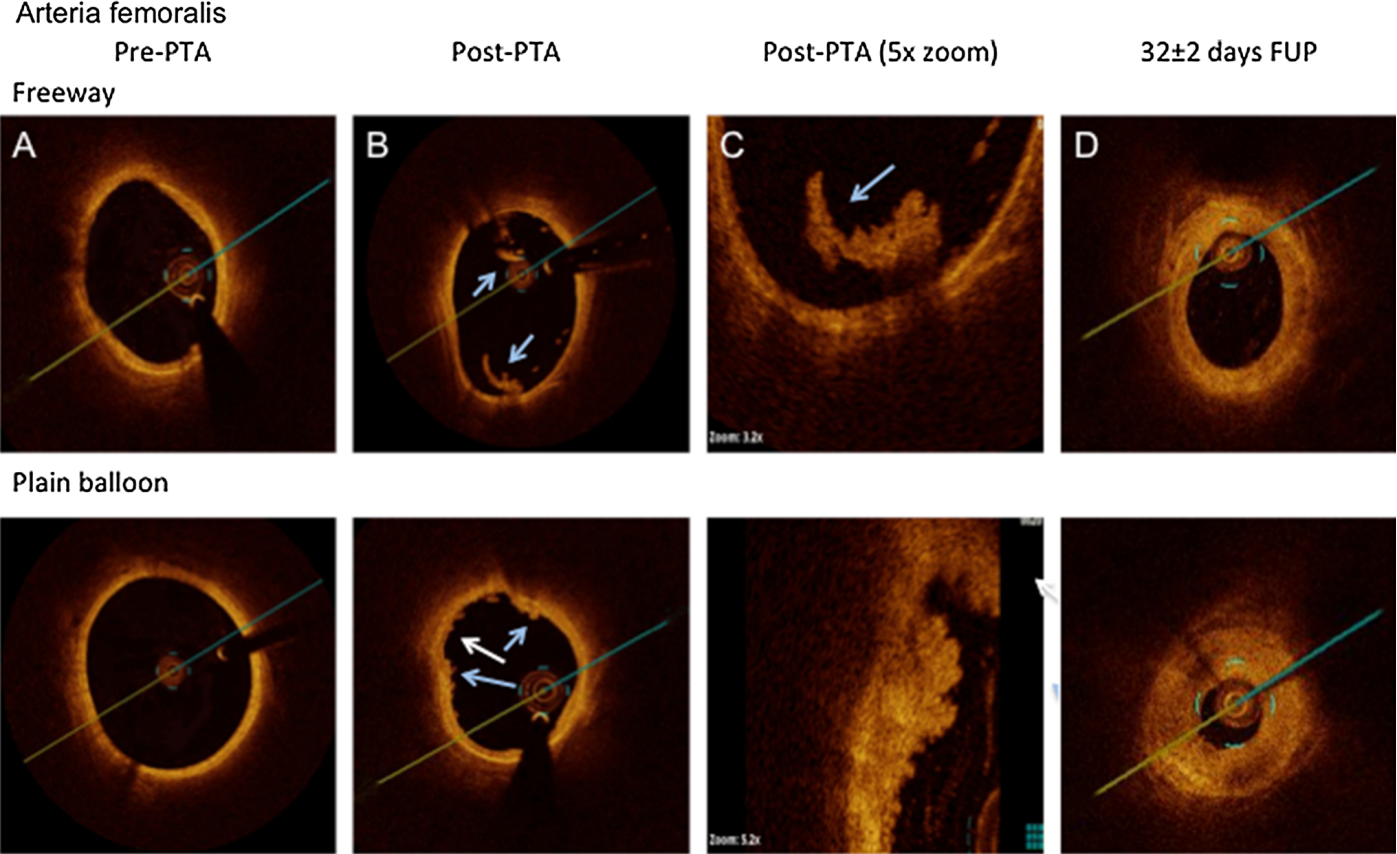
OCT images of the femoral arteries before and after percutaneous transluminal angioplasty (PTA) with Freeway™ (*upper* *panel*) and plain balloon (*bottom* *panel*) and at follow-up (FUP). **a** Pre-PTA: Image taken before percutaneous transluminal angioplasty (Pre-PTA) of the native femoral artery. **b** Post-PTA: Image taken after balloon overstretch injury of femoral arteries, showing white platelet-rich thrombi (*blue arrow*) at the site of intimal rupture and intraluminally. Small attaching white thrombi (*white arrow*). **c** Post-PTA (×5 magnification) of a white intraluminal thrombus (*blue arrow*) partially attaching to the vessel wall with an irregular structure and shadowing (*upper panel*). The *bottom panel* shows severe rupture of the intima and media (*white arrow*) with attaching white thrombi. **d** 32 ± 2 days FUP: Medium- and high-grade obstruction of the femoral arteries at 32 ± 2 days after balloon overstretch injury. Shrinkage of the artery suggests severe constrictive remodeling after plain balloon dilation (Color figure online)

**Fig. 5:**
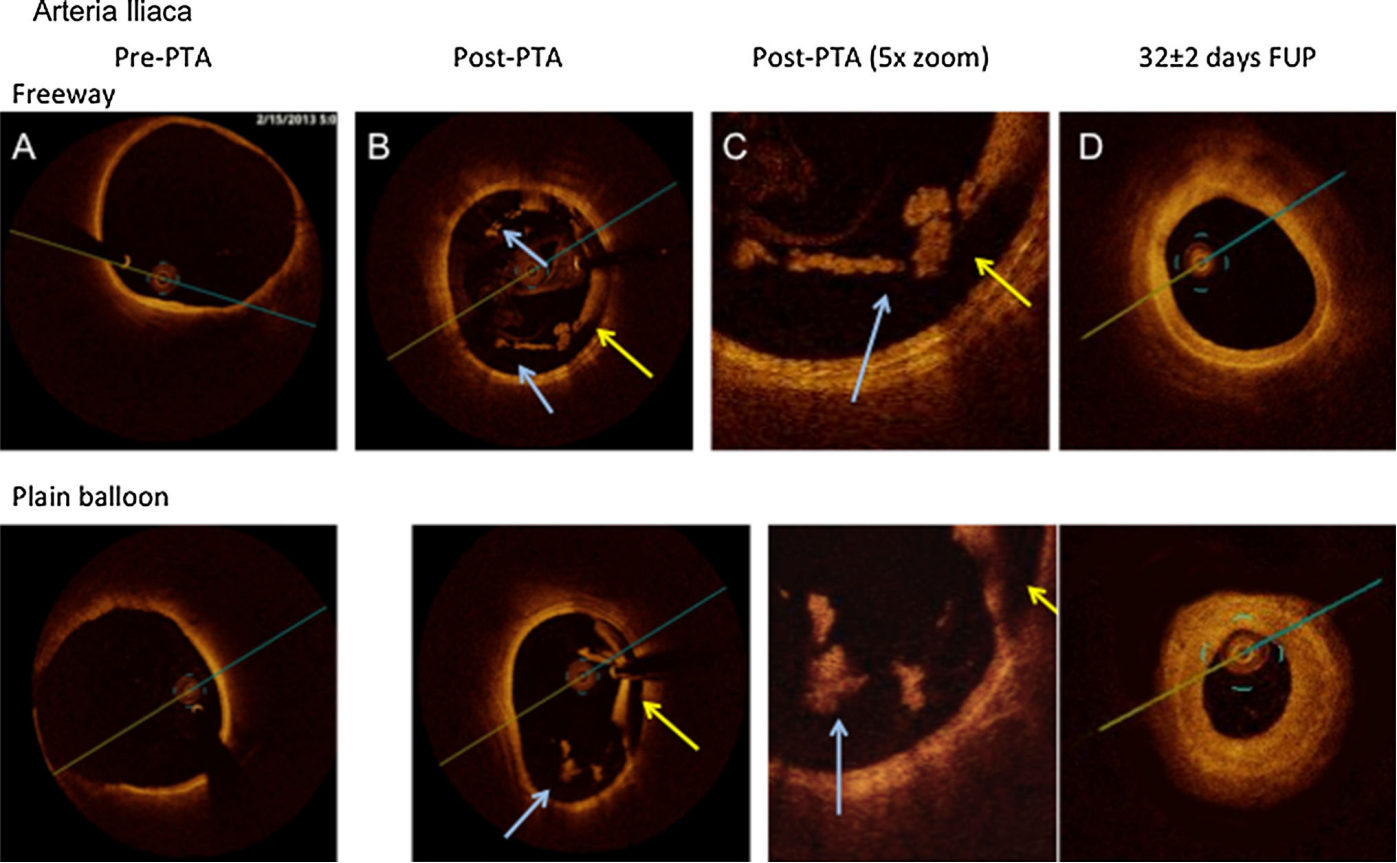
OCT images of the iliac arteries pre- and post-percutaneous transluminal angioplasty (PTA) and at follow-up (FUP)**. a** Pre-PTA: OCT image of a native iliac artery showing a blurred contour away from the imaging probe, highlighting the difficulty of correctly imaging large arteries. **b** Post-PTA: Post-balloon overstretch injury of iliac arteries with large dissections (*yellow arrows*) and platelet-rich white thrombi, both parietal and intraluminal (*blue arrows*).** c** Post-PTA (×5 magnification) of vessel wall dissection (*yellow arrow*) and white intraluminal thrombus (*blue arrow*) partially attached to the vessel wall with irregular structure and shadowing. **d** 32 ± 2 days FUP: Partial medium-grade obstruction of the iliac arteries 32 ± 2 days after balloon overstretch injury. Artery shrinkage suggests constrictive remodeling of the iliac vessels due to neointima development, but to a lesser extent than in the femoral arteries (Color figure online)

Overstretch balloon dilation led to severe vessel injury in each case, as confirmed by angiography, OCT, and histology (Figs. 3, 4, 5). OCT images showed severe vessel wall injury after overstretch balloon dilation, including dissection, and parietal and intraluminal floating thrombi, but without acute artery closure. At 1 h after balloon dilation, fluorescent staining of the femoral arterial cross-sections showed cell-rich (plain balloon) or cell-poor (Freeway™) intraluminal thrombus formation, and massive expression of tumor necrosis factor-alpha by the inflammatory cells and platelets (Figs. 3, 4, 5).

### Paclitaxel accumulation in the peripheral vessel wall

**Fig. 6:**
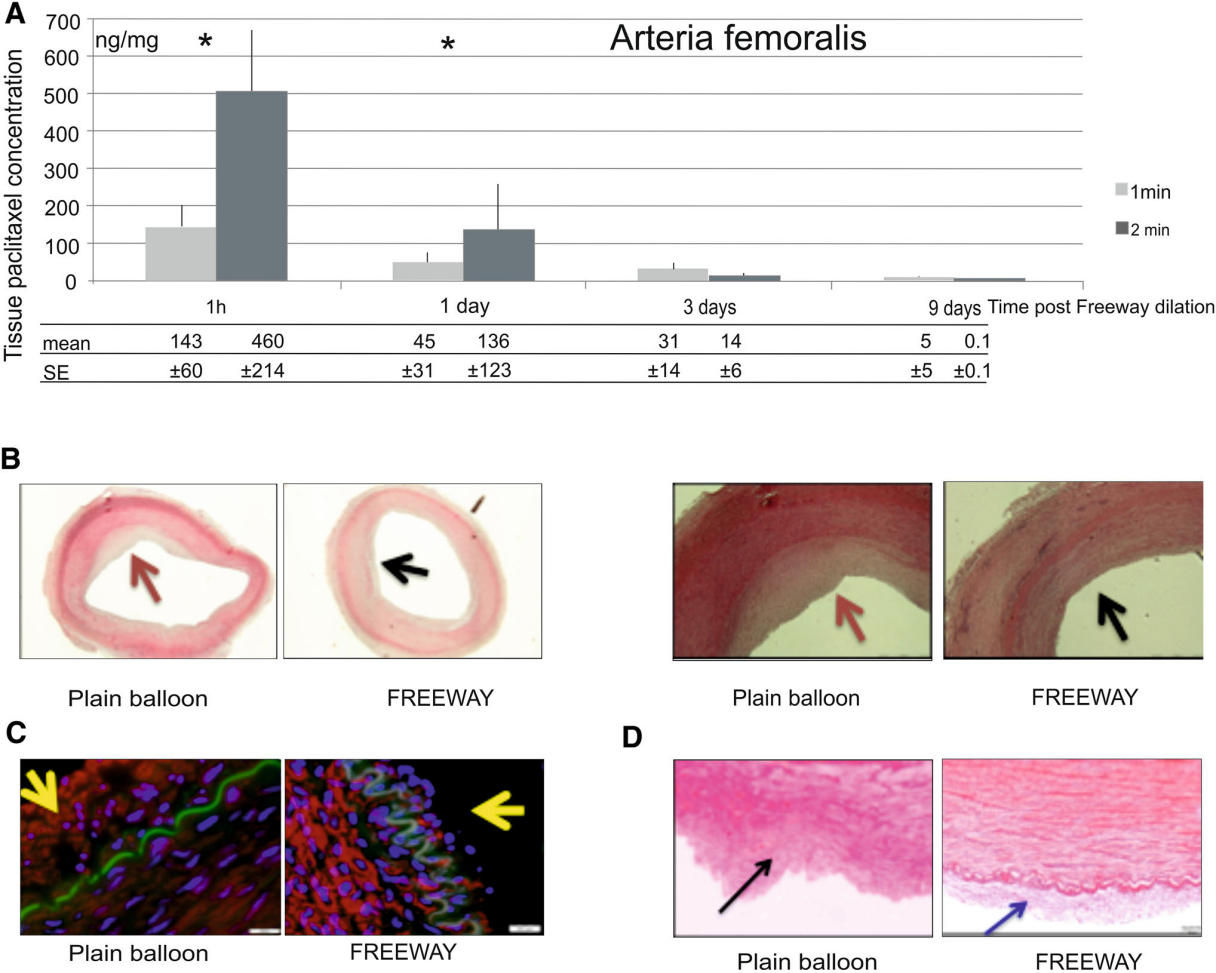
Pharmacokinetic and histologic studies of the femoral arteries. **a** Arteria femoralis tissue paclitaxel concentrations at different follow-up times after a 1- or 2-min balloon inflation time. **b** Cross-sections of porcine femoral arteries at 32 ± 2 days after overstretch dilation. Uneven distribution of neointimal hyperplasia (*red arrow*) after dilation with the plain balloon, and a circumferential small degree of neointima (*black arrow*) after dilation with the Freeway™ (*left two panels*). Arterial Sects. (×4 magnification) showing neointimal hyperplasia following dilation with either the plain balloon or Freeway™ (*right two panels*). Hematoxylin–eosin staining. **c** Invasion of smooth muscle cells showing positive alpha actin staining of femoral arteries into the intima layer (*yellow arrow*), with substantially fewer cells in the intima in the Freeway™ group. **d** Fibrin deposition in the intima (*black arrow*) with a disintegrated internal elastic lamina structure and no clear intersection between intimal hyperplasia and tunica media after plain balloon overstretch injury (*left*). In contrast, after Freeway™ dilation, thin neointimal hyperplasia was observed (*blue arrow*) (*right*). Picrosirius red staining, ×8 magnification (Color figure online)

**Fig. 7:**
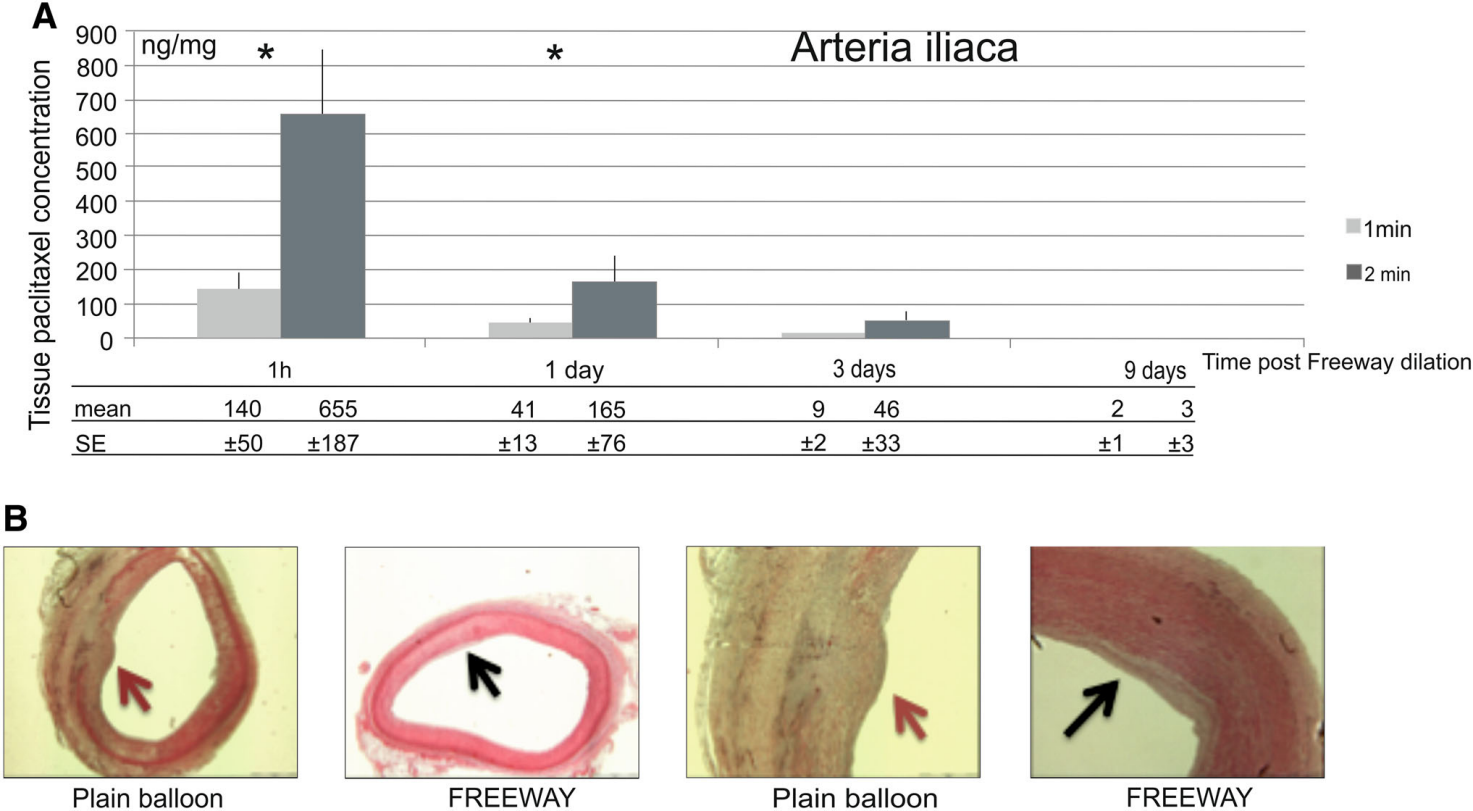
Pharmacokinetic and histologic studies. **a** Arteria iliaca tissue paclitaxel concentration at different follow-up times after a 1- or 2-min balloon inflation time. **b** Cross-sections of porcine iliac arteries at 32 ± 2 days after overstretch dilation, showing uneven distribution of neointimal hyperplasia (*red arrow*) after dilation with a plain balloon, and a circumferential small degree of neointima (*black arrow*) after Freeway™ dilation (*left two panels*). Arterial Sects. (×4 magnification) with neointimal hyperplasia after dilation with either plain balloon or Freeway™ (*right two panels*). Focal neointimal hyperplasia at the site of rupture of the internal elastic membrane, with inflammatory cells in the media and adventitia (*red arrow*). Minimal neointimal hyperplasia in arteries dilated with Freeway™ (*black arrow*). Hematoxylin–eosin staining (Color figure online)

Compared with 1-min dilation, the 2-min inflation time was definitively more effective in terms of tissue retention of the drug in both arteries (Figs. 6, 7). Therefore, we used the 2-min balloon inflation time in our vascular response and remodeling studies, which is also the more commonly used inflation time in human peripheral interventions.

Following 2-min balloon inflation of the femoral arteries, the paclitaxel concentration at the 1-h follow-up was 36.9 ± 17.8 ng/mg in the proximal reference segments and 17.0 ± 6.3 ng/mg in the distal reference segments. As expected, the plasma paclitaxel concentration decreased with time after DCB use. At 5 and 10 min after a 1-min balloon inflation, the paclitaxel concentrations decreased from 9.2 ± 4.5 to 0 ng/mL. At 5, 10, 20, and 60 min after a 2-min balloon inflation, the paclitaxel concentrations were 14.6 ± 6.8, 10.2 ± 4.9, 7.7 ± 3.1, and 0.6 ± 0.4 ng/mL, respectively. The percentages of paclitaxel remaining on the balloon surface after the procedures were 53.2 ± 9.8 and 30.9 ± 4.3 % following 1- and 2-min dilation of the femoral arteries, respectively.

### Vessel remodeling study

Quantitative peripheral angiography revealed that Freeway™ dilation was associated with a significant percentage of decrease in the stenosis diameter of the femoral arteries, and with a trend toward less stenosis of the iliac arteries (Tables 1, 2). Among ten selected OCT images, the intra- and inter-observer variability values were r = 0.95 and r = 0.91 (*P* < 0.001), respectively. At 32 ± 2 days, OCT imaging revealed that Freeway™ was associated with a significantly lower percentage area of stenosis in both the femoral and iliac arteries (Tables 3, 4). With the use of plain balloons, OCT indicated vessel constriction (Figs. 4, 5). Quantitative OCT images revealed a remodeling index of <1 in femoral arteries dilated with a plain balloon, suggesting constrictive remodeling (Tables 3, 4). No meaningful arterial remodeling was observed in the elastic-type iliac arteries (Tables 3, 4).

**Table 1:** Quantitative angiographic parameter of the femoral arteries 32 ± 2 days follow-up (FUP)

Femoral arteries	Plain balloon (n = 10)	Freeway™ balloon (n = 10)
Lumen diameter pre-dilation (mm)	5.1 ± 0.3	5.2 ± 0.5
Lumen diameter post-dilation (mm)	6.6 ± 0.6	6.7 ± 0.6
Balloon/artery ratio	1.31 ± 0.08	1.29 ± 0.11
Lumen diameter at FUP (mm)	4.6 ± 0.7	5.2 ± 0.6
Vessel diameter at FUP (mm)	6.2 ± 0.6	6.3 ± 0.7
%Diameter stenosis at FUP (%)	25.8 ± 10.7	17.6 ± 12.2*

* *P* < 0.05 between plain balloon and Freeway™

**Table 2:** Quantitative angiographic parameters of the iliac arteries 32 ± 2 days follow-up (FUP)

Iliac arteries	Plain balloon (n = 10)	Freeway™ balloon (n = 10)
Lumen diameter pre-dilation (mm)	7.4 ± 0.6	7.5 ± 0.6
Lumen diameter post-dilation (mm)	9.7 ± 0.6	9.7 ± 0.6
Balloon/artery ratio	1.31 ± 0.10	1.31 ± 0.08
Lumen diameter at FUP (mm)	7.0 ± 0.5	7.2 ± 0.6
Vessel diameter at FUP (mm)	8.8 ± 0.8	8.7 ± 0.6
%Diameter stenosis at FUP (%)	20.5 ± 4.3^+^	17.2 ± 4.2

^+^ *P* < 0.1

**Table 3:** OCT parameters of the femoral arteries at baseline (pre-dilation), and at 32 ± 2 days follow-up (n = 10 arteries of 5 animals)

Femoral arteries	Plain balloon	Freeway™ balloon
Baseline		
Minimal lumen diameter (mm)	5.4 ± 0.3	5.4 ± 0.7
Vessel diameter (mm)	5.6 ± 0.4	5.6 ± 0.7
Minimal lumen area (mm^2^)	21.3 ± 3.2	20.1 ± 5.8
IEM area (mm^2^)	22 ± 3.3	21.1 ± 5.9
Follow-up		
Minimal lumen diameter (mm)	3.1 ± 0.3	4.8 ± 0.5*
Vessel diameter (mm)	5.0 ± 1.2	5.8 ± 0.7
Minimal lumen area (mm^2^)	9.7 ± 1.5	17.8 ± 3.9*
Intima area (mm^2^)	7.3 ± 1.7	4.3 ± 2.4*
IEM area (mm^2^)	17.0 ± 3.1	22.1 ± 5.5
Percent area stenosis (%)	42.8 ± 3.1	18.7 ± 10.1*
IEM area of reference segment (mm^2^)	21.3 ± 1.9	21.8 ± 5.4
Remodeling index	0.80 ± 0.15	1.02 ± 0.03*

IEM internal elastic membrane

* *P* < 0.05 between plain balloon and Freeway™

**Table 4:** OCT parameters of the iliac arteries at baseline (pre-dilation), and at 32 ± 2 days follow-up (n = 10 arteries of 5 animals)

Iliac arteries	Plain balloon	Freeway™ balloon
Baseline		
Minimal lumen diameter (mm)	5.9 ± 0.9	5.9 ± 0.5
Vessel diameter (mm)	6.0 ± 0.8	6.1 ± 0.4
Minimal lumen area (mm^2^)	24.0 ± 4.9	23.8 ± 6.2
IEM area (mm^2^)	24.8 ± 4.6	24.6 ± 6.3
Follow-up		
Minimal lumen diameter (mm)	5.2 ± 0.9	5.5 ± 0.9
Vessel diameter (mm)	6.2 ± 1.0	6.3 ± 0.5
Minimal lumen area (mm^2^)	18.5 ± 6.8	22.0 ± 7.3
Intima area (mm^2^)	8.2 ± 2.1	3.5 ± 1.6*
IEM area (mm^2^)	26.6 ± 6.0	25.5 ± 6.3
Percent area stenosis (%)	32.4 ± 13.4	14.9 ± 8.6*
IEM area of reference segment (mm^2^)	26.3 ± 2.1	25.5 ± 6.0
Remodeling index	1.01 ± 0.18	1.00 ± 0.03

IEM internal elastic membrane

* *P* < 0.05 between plain balloon and Freeway™

**Fig. 8:**
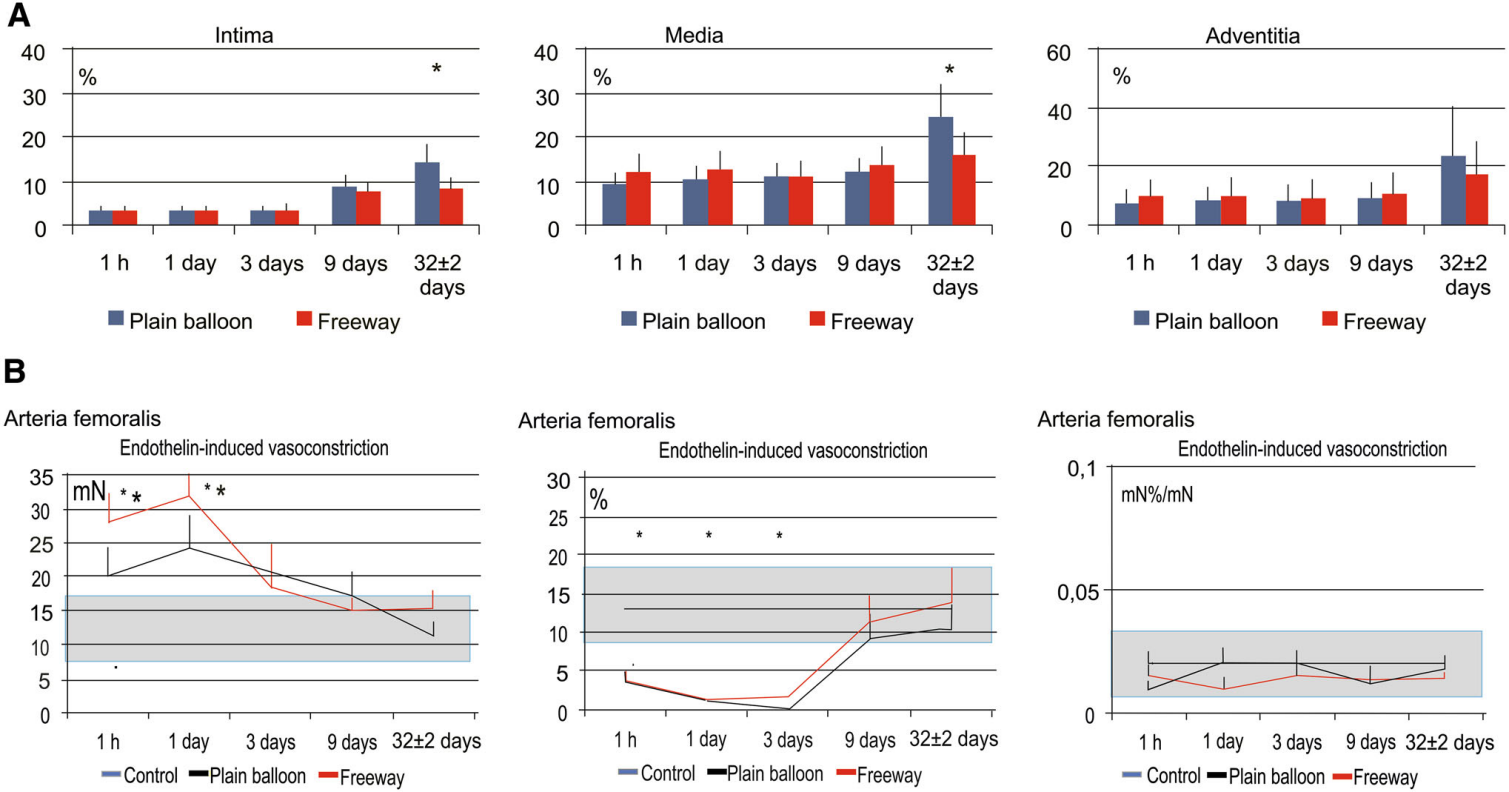
Time-dependent development of arterial tissue fibrosis (intima) and fibrous collagen (media) after balloon dilation, and the vasomotor response (vasoconstriction and vasodilation) of the femoral arteries. **a** Time-dependent increase in arterial tissue fibrosis/collagen in the different femoral artery layers (intima, tunica media, and adventitia) after dilation with the plain balloon or Freeway™ paclitaxel-coated balloon. **b** Endothelin-induced contraction of the femoral artery (*left*). Susceptibility to contraction at 1 day post-dilation with either type of balloon, followed by rapid normalization. Profound injury of the endothelium-dependent vasodilatory reaction, with no difference between the two balloon types (*mid*). No change in endothelium-independent vasodilation (*right*). The *gray* range represents the normal value ±1 standard deviation. *P < 0.05 between balloon and control, ^+^ *P* < 0.05 between Freeway™ and plain balloon. Number of arteries listed in Fig. 1

**Fig. 9:**
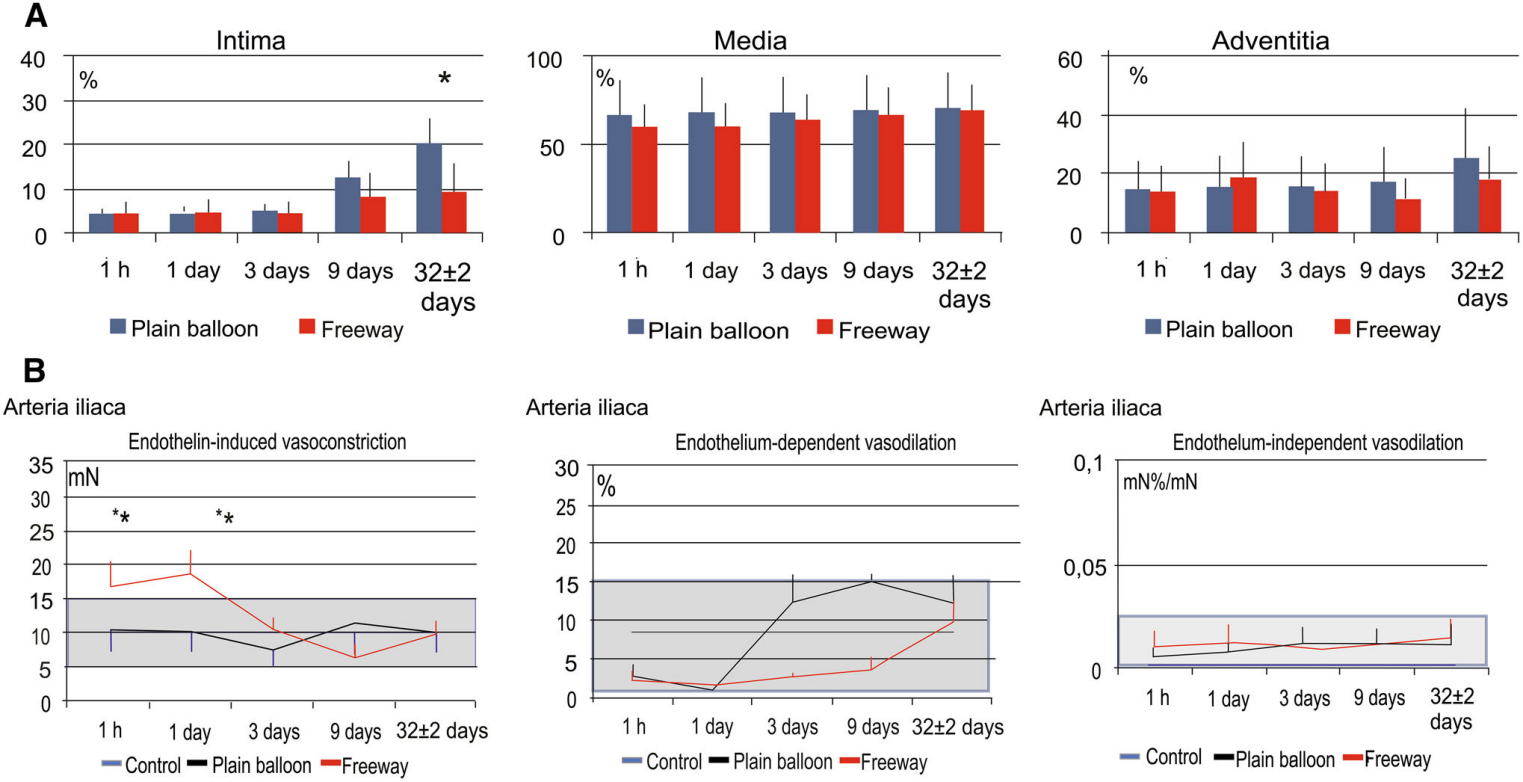
Time-dependent development of arterial tissue fibrosis/collagen after balloon dilation, and vasomotor response (vasoconstriction and dilation) of the iliac arteries. **a** Time-dependent increase in arterial tissue fibrosis/collagen in the different iliac artery layers (intima, tunica media, and adventitia) after dilation with a plain balloon or Freeway™ paclitaxel-coated balloon. **b** Endothelin-induced iliac artery contraction (*left*). Susceptibility to contraction at 1 day post-dilation with the Freeway™ drug-coated balloon, followed by rapid normalization. Normal endothelium-dependent and endothelium-independent vasodilatory reaction after use of either balloon types. The gray range represents the normal value ±1 standard deviation. *P < 0.05 between balloon and control, ^+^ *P* < 0.05 between Freeway™ and plain balloon. Number of arteries listed in Fig. 1

Histopathological analyses showed similar inflammation and injury scores between the two groups. Both device groups showed complete endothelialization in femoral arteries. In the iliac arteries, endothelialization was complete in the plain balloon group and was 95 ± 5 % with the Freeway™ balloon (Tables 5, 6). No vessel calcification was observed. Histomorphometric analyses of both arteries revealed that the Freeway™ group showed a significantly larger lumen area, smaller neointimal area, and lower percentage area of stenosis compared to these values in the plain balloon group (Tables 7, 8). Alpha-actin staining revealed invasion of smooth muscle cells into the intima in the femoral arteries after plain balloon use, in contrast with the Freeway™-dilated arteries (Fig. 6). Additionally, arterial wall exposure to paclitaxel inhibited fibrin deposition in the tunica intima and media in the femoral arteries (Fig. 8), and in the intima in the iliac arteries (Fig. 9).

**Table 5:** Histopathological parameters of femoral arteries 32 ± 2 days after balloon dilation with Freeway™ or plain balloon

Femoral arteries	Plain balloon (n = 10)	Freeway™ balloon (n = 10)
Inflammation score	0.30 ± 0.22	0.32 ± 0.13
Hemorrhage	0	0
Necrosis	0	0
Medial injury	0.89 ± 0.11	0.93 ± 0.25
Endothelialization complete	100 %	100 %
Injury score	0.92 ± 0.64	0.90 ± 0.67
Calcification	0	0

No significant differences between the groups

**Table 6:** Histopathological parameters of the iliac arteries 32 ± 2 days after balloon dilation with Freeway™ or plain balloon

Iliac arteries	Plain balloon (n = 10)	Freeway™ balloon (n = 10)
Inflammation score	0.42 ± 0.17	0.45 ± 0.36
Hemorrhage	0	0
Necrosis	0	0
Medial injury	0.86 ± 0.22	0.79 ± 0.13
Endothelialization complete	100 %	95 ± 5 %
Injury score	1.0 ± 0.68	1.1 ± 0.83
Calcification	0	0

No significant differences between the groups

**Table 7:** Histomorphometric parameters of the femoral arteries 32 ± 2 days after balloon dilation with either plain balloon or Freeway™ drug-coated balloon

Femoral arteries	Plain balloon (n = 10)	Freeway™ balloon (n = 10)
Lumen area (mm^2^)	8.1 ± 1.7	10.2 ± 1.1*
Neointimal area (mm^2^)	3.7 ± 1.8	0.9 ± 0.1*
IEL area (mm^2^)	11.7 ± 2.2	11.1 ± 1.0
Media area (mm^2^)	6.6 ± 4.5	10.2 ± 2.8
EEL area (mm^2^)	18.3 ± 2.6	21.3 ± 2.1
%Area stenosis (%)	30.5 ± 14.2	8.3 ± 1.8*
Maximal neointimal thickness (mm)	2.9 ± 0.7	0.67 ± 0.32*
EEL area of the reference segment (mm^2^)	21.8 *±* 2.6	21.5 ± 1.8
Remodeling index	0.84 ± 0.04	1.01 ± 0.07*

IEL internal elastic lamina, EEL external elastic lamina

* *P* < 0.05 between plain balloon and Freeway™

**Table 8:** Histomorphometric parameters of the iliac arteries 32 ± 2 days after balloon dilation with either plain balloon or Freeway™ drug-coated balloon

Iliac arteries	Plain balloon (n = 10)	Freeway™ balloon (n = 10)
Lumen area (mm^2^)	10.3 ± 1.0	12.2 ± 1.5*
Neointimal area (mm^2^)	4.1 ± 0.7	2.3 ± 0.9*
IEL area (mm^2^)	14.4 ± 0.4	14.6 ± 1.2
Media area (mm^2^)	8.7 ± 2.3	9.8 ± 3.9
EEL area (mm^2^)	23.1 ± 1.9	24.3 ± 4.8
%Area stenosis (%)	28.3 ± 5.6	16.0 ± 6.3*
Maximal neointimal thickness (mm)	2.0 ± 0.2	0.94 ± 0.83*
EEL area of the reference segment (mm^2^)	23.8 ± 2.5	24.3 ± 4.8
Remodeling index	0.97 ± 0.03	1.01 ± 0.01

IEL internal elastic lamina, EEL external elastic lamina

* *P* < 0.05 between plain balloon and Freeway™ in iliac arteries

### Physiological response of the peripheral arteries to balloon dilation

Enhanced endothelin-induced vasoconstriction in the Freeway™-treated peripheral arteries was more pronounced in the femoral arteries than in the iliac arteries, and showed rapid normalization at 3 days post-intervention (Figs. 8, 9). The endothelium-dependent vasodilator reaction was profoundly impaired during the first 3 days in the femoral vessels but not in the iliac arteries, with no difference between the two balloon types. Endothelium-independent vasodilation did not change after balloon injury in the femoral or iliac arteries (Figs. 8, 9).

## Discussion

Our present study results provide novel data with translational value regarding the use of DCB in clinical practice. We report that arterial dilation with the DCB Freeway™ inhibited fibrin accumulation in the tunica intima and media of the femoral arteries, leading to significantly less constrictive remodeling of the injured vessel. However, tissue uptake of the drug into the vessel wall after percutaneous intervention resulted in a predisposition to vessel constriction in both the iliac and femoral arteries, as well as impaired endothelium-dependent vasodilation of the femoral arteries. Our present report also includes the application of high-resolution OCT imaging for in vivo display of peripheral vascular injury following the use of an intravascular device, which demonstrated non-flow-limiting dissection and thrombus formation with an inflammatory reaction. Finally, we report the pathophysiological differences between femoral and iliac arteries in response to intravascular intervention using either the plain balloon or DCB (Table 9).

**Table 9:** Currently available drug-coated balloons for peripheral artery disease

Company	Device name	CE-certificate	FDA approval	Coating	Drug dose (ug/mm^2^)
Aachen resonance holding (Luxembourg)	Elutax	Yes (2013)	No	PTX-Hydrogel	3.0
BARD (US)	Lutonix	Yes (2011)	Yes (2014)	PTX-polysorbate/Sorbitol	2.0
Bayer schering pharma AG (Germany)	Cotavance	Yes (2011)	No	PTX-Iopromide	3.0
Biotronik AG (Germany)	Passeo-18 Lux	Yes (2014)	No	PTX-BTHC	3.0
Cardionovum GmbH (Germany)	Legflow	Yes (2011/12)	No	PTX-Shellac	3.0
Cook medical (US)	Advance 18 PTX	Yes (2011)	No	PTX	3.0
Covidien (US)	Stellarex	No	No	PTX	2.0
Eurocor GmbH (Germany)	Freeway	Yes (n/a)	No	PTX-Shellac	3.0
Medtronic vascular (Switzerland)	In.pact admiral	Yes (2009)	Yes (2014)	PTX-Urea	3.0
Boston scientific (US)	Ranger	Yes	No	PTX-BTHC	2.0
iVascular (Spain)	Luminor	Yes	No	PTX	3.0

### Tissue and plasma drug levels after balloon catheter-derived delivery: comparison with data from the literature

Our present results showed that use of the Freeway™ DCB led to paclitaxel accumulation in both the femoral and iliac arteries, and the longitudinal distribution of this drug both proximal and distal to the balloon dilation site. Yazdani et al. previously reported much lower paclitaxel levels in the arteria femoralis tissue of healthy domestic swine following their use of the Lutonix drug-coated balloon (Lutonix Inc., New Hope, MN). However, in their study, the femoral arteries were dilated without overstretch injury for an inflation time of only 30 s [34]. In another study, Buszmann et al. measured paclitaxel levels at 1 h after 30-s dilation with the Cotavance DCB (MEDRAD Interventional, Indianola, PA) in hypercholesterolemic domestic pigs with in-stent restenosis of the peripheral arteries, and reported paclitaxel tissue uptake of <50 ng/mg tissue [17]. Comparatively, our present study showed paclitaxel uptake of 143 ± 60 and 460 ± 214 ng/mg femoral artery tissue at 1 h after balloon dilation with a 1- or 2-min inflation time, respectively. Granada et al. [16] reported that histology, but not quantitative angiography, is sufficiently sensitive to determine the effect of a zotarolimus-coated balloon for restenosis prevention. Like in our study, Kolachalama et al. demonstrated that the tissue drug level of zotarolimus in peripheral arteries was dependent on the balloon inflation time [35].

In contrast with previously published peripheral DCB studies, in our present study, we separately analyzed the iliac and femoral arteries because these arteries differ in structure and in their physiological responses to injury (as shown in our study). We also applied optical imaging to examine the morphological effects of balloon inflation in the femoro-iliac arteries. OCT images of peripheral arteries revealed severe post-dilation vessel injury, including dissections and luminal thrombi, and proved to be more sensitive than angiography for the assessing lumen morphology and dimensions, similar to histology.

It appears that a long presence and high concentration of the drug in the arterial wall are crucial for inhibition of neointimal proliferation and restenosis [36, 37]. In our study, the paclitaxel tissue level was maintained over 9 days—beyond occurrence of the initial thrombotic and inflammatory processes following vessel trauma from endovascular interventions, and beyond the smooth muscle cell migration and proliferation from the media and adventitia. Longer presence of an antiproliferative drug—especially one with a narrow therapeutic window, like paclitaxel—could result in vascular toxicity, including ectasia of the treated vessels [21]. Our present results showed that a 1-min balloon inflation time in peripheral arteries resulted in an artery tissue paclitaxel level similar to that previously observed in coronary arteries [31, 32]. Due to the potential for untoward cardiac effects from prolonged ischemia, a longer balloon inflation time is not desirable in coronary arteries. Paclitaxel is released into systemic circulation immediately after balloon inflation. The use of a 2-min balloon inflation time led to a gradual decrease in the plasma drug level, corresponding to the plasma paclitaxel clearance half-life [38]. Dose-finding clinical investigations show that a plasma concentration below 6.3 µmol/L (5400 ng/mL; well above the 15 ng/mL maximal level in our study) is not associated with any systemic toxicity [39, 40].

### Vascular remodeling study

Paradoxical arterial (or constrictive) remodeling was first described in femoral arteries by Pasterkamp et al. [41]. Our present experiments revealed that paclitaxel accumulation in the femoral arterial wall inhibited fibrin deposition in the intimal and medial layers. Paclitaxel did not substantially influence the amount of fibrous collagen in the tunica media of the iliac arteries, but it did reduce fibrin deposition in the intima. Thus, the higher post-intervention lumen area may be explained not only by inhibition of smooth muscle cell migration into the intima, but also by prevention of constrictive remodeling. While tissue drug levels are published for other DCB systems, such vessel remodeling and pathophysiological studies have not yet been performed for other DCBs.

### Pathophysiological study

Our physiological investigation revealed that tissue drug level was clearly associated with increased vasoconstrictor tone of the affected vessel. This association was more pronounced in the femoral arteries than in the iliac vessels during the first days following overstretch injury. Dilation with the DCB led to increased vasoconstrictive response in both the iliac and femoral arteries, and notably in vessels with injury scores similar to those of arteries dilated with the plain balloon. Thus, the overstretch injury (denudation) of the arteries was not the only cause of this pathological reaction.

Vascular procedures induce a reflexive, mechanically induced vasospasm. In contrast to the coronary arteries [29], the intact control peripheral vessels showed much lower endothelium-dependent vasodilator capacity. Notably, endothelium-independent (muscular layer) vasodilation was unaffected by the balloon overstretch injury, likely due to the high fibrous collagen tissue content. The results of our previous physiological studies in coronary arteries [29] together with our current findings in peripheral vessels, appear to indicate that the peripheral arteries are much less reactive with regards to contraction and to endothelium-dependent and -independent vasodilation. This fact may be particularly important if coronary devices are tested in rabbit iliac arteries.

### Practical application and translational nature of the study

The Freeway™ DCB is commercially available in Europe and in several non-USA countries for human peripheral artery interventions. However, animal studies of this device still provide a unique opportunity to examine the reaction of the host tissue; to investigate tissue pathology and inflammation; to study drug retention, delivery, kinetics, and toxicology; to assess systemic drug disposition or other harmful effects related to the application of drugs and/or a foreign body; and to further explore their mechanisms of action. These data acquired from animal research can directly lead to improvements of technological concepts and to refinements of intravascular devices. In contrast, human trials typically focus on the safety and efficacy of an interventional therapy, without attaining new information regarding the underlying biology.

Accordingly, in our present animal model of the use of peripheral intravascular DCB, we demonstrated that the drug coating on an intravascular balloon surface did not cause delayed re-endothelialization in peripheral arteries. Thus, in contrast with drug-coated stents, the use of DCB could reduce the duration of dual antiplatelet therapy [36], which is of clinical interest. In cases of non-flow-limiting dissection, the use of a DCB is apparently sufficient to prevent restenosis without provisional stenting in peripheral arteries, thus avoiding stent fractures or foreign body reactions. Moreover, the use of DCBs for peripheral artery dilation prevents unfavorable constrictive remodeling, thereby inhibiting disease progression. It is also notable that the impaired vasoreactivity caused by drug deposition in the arterial wall requires more aggressive post-intervention vasodilatory treatment.

### Limitations

Percutaneous intervention of healthy porcine peripheral arteries cannot mimic the peripheral intervention of atherosclerotic human arteries, and is not entirely comparable with the injury model of familial hypercholesterolemic swine [16]. It was recently proposed that deep acute endothelium injury in hypercholesterolemic swine could be used as an animal model for device assessment [16]. However, the current guidelines for performing pre-clinical tests in coronary and peripheral arteries do not recommend the use of diseased porcine models [34]. Nevertheless, in our present study, visible injury of the peripheral arteries was confirmed by angiography and OCT following gross overdilation. It should also be noted that the pathophysiological response of the denuded artery [16] influences the endothelial uptake of locally delivered drugs. Further limitations of this study are described in the Supplemental Material.

## Conclusions

Our present results demonstrated the safety and efficacy of the Freeway™ paclitaxel-coated balloon in a preclinical model of overstretch injury in peripheral arteries. The observed beneficial effects of the Freeway™ balloon may be attributed to inhibition of smooth muscle cell migration and neointimal hyperplasia development, as well as prevention of fibrosis of the intima and media, resulting in less constrictive vessel remodeling. We also showed that higher arterial wall paclitaxel concentration was associated with local vasoconstriction and impaired endothelium-dependent vasodilation. Furthermore, our findings show that although high-resolution OCT imaging is technically challenging, it was useful for in vivo display of peripheral vascular injury after intravascular device usage, demonstrating non-flow-limiting dissection and thrombus formation with inflammatory reaction in peripheral arteries.

## Electronic supplementary material

Below is the link to the electronic supplementary material.

Supplementary material 1 (DOCX 158 kb)
