# Supplementary material for: Coating of intravascular balloon with paclitaxel prevents constrictive remodeling of the dilated porcine femoral artery due to inhibition of intimal and media fibrosis
Source: J Mater Sci Mater Med. 2016 Jul 7;27:131. doi: 10.1007/s10856-016-5737-y (PMC4937086; doi:10.1007/s10856-016-5737-y)
Supplement: Supplementary file 1 — Supplementary material 1 (DOCX 158 kb) [file 10856_2016_5737_MOESM1_ESM.docx]

**Coating of intravascular balloon with paclitaxel prevents development of constrictive remodeling after dilation of porcine femoral arteries due to inhibition of intimal and media fibrosis**

***Supplemental Materials and Methods***

*Supplemental Method, Animal preparation*

Before the peripheral intervention, the pigs received an intramuscular injection of 12 mg/kg ketamine hydrochloride, 1 mg/kg xylazine, and 0.04 mg/kg atropine, followed by inhalation anesthesia with isoflurane and O_2_ via a mask. Upon reaching deep anesthesia, pigs were intubated and received an anesthetic gas mixture of 1.5–2.5 vol% isoflurane, 1.6–1.8 vol% O_2_, and 0.5 vol% N_2_O. Blood pressure, O_2_ saturation, and electrocardiogram were continuously monitored throughout the protocol.

Surgical preparation of the left carotid artery was performed under sterile conditions, followed by insertion of a 6F introduction sheath. After intra-arterial administration of 200 IU/kg of unfractionated heparin, a 6F right coronary catheter (Medtronic Inc.) was placed into the abdominal aorta, and selective angiographies of the left and right iliac and femoral arteries were performed.

*Supplemental Method, Study design*

To determine the tissue paclitaxel accumulation, a pharmacokinetic study was performed in 29 pigs, of which 14 and 15 underwent 1- and 2-min paclitaxel–DCB inflation, respectively. For the vascular response (vasodilation/constriction) study, the pig arteries were randomly treated with either Freeway DCB or plain balloon. In vitro vascular pathophysiological measurements were done in 60 arteries of 15 pigs randomized to Freeway™ or plain balloon dilation or to control, with 1 h and then 1, 3, 9, and planned 4 weeks (32±2 days) of follow-up (FUP).

Ten pigs underwent randomized vessel remodeling with additional safety and efficacy studies with 32±2 days of FUP using Freeway™ or plain balloon overstretch injury. Before and immediately after balloon overstretch dilation and at FUP, OCT (St. Jude Medical, St. Paul, Minnesota; San Diego, California) imaging was performed in five pigs. Quantitative OCT and angiographic measurements of the vessel dimensions were performed to assess stenosis grade and vessel remodeling.

*Supplemental Method, Vascular biology study measuring vasocontrictor tone and endothelium-dependent and –independent vasodilation*

The dilated sections of the peripheral arteries (femoral and iliac) were freshly prepared, and the fat and connective tissues were removed. Ring segments 4 mm in length were mounted in a temperature-controlled (37^°^C) tissue bath containing a modified Krebs–Henseleit buffer solution. Briefly, the isometric circular wall tension of the vessels was measured using the organ bath chamber method and modified myography. Each segment was suspended between two L-shaped metal pins (0.4 mm in diameter) connected to the measurement system of the myograph.

Following the establishment of the steady-state condition, the vessels were contracted with 30 nM endothelin-1. Endothelium-dependent vasodilation was provoked by a bolus application of substance P into the organ bath with a final concentration of 1 nM. After the stable condition was restored, the sensitivity of smooth muscle to external NO was measured by addition of 4 mM sodium nitroprusside.

Vasoconstriction, corresponding to media injury and/or endothelial dysfunction in regulation of vascular tone, was expressed as mN. The endothelium-dependent and –independent vasodilation (stiffness of vascular smooth muscle after overstretch injury of PCI) was expressed as % change in steady-state level contraction, and in mN/s/mN units, respectively.

*Supplemental Method, Quantitative angiographic and OCT analysis*

Quantitative angiographic measurements were performed with a computer-assisted quantitative coronary arteriographic edge-detection algorithm (for cineangiograms: Cardiovascular Measurement System, Medis, the Netherlands; for CD-ROM: ACOMPC, Siemens, Germany). Measurements were made of the minimal lumen and reference vessel diameters of the dilated and proximal and distal reference segments, followed by calculation of the % diameter stenosis.

Quantitative OCT measurements were performed with LightLab Imaging software using the Guidelines for OCT Imaging and Reporting. The sites of analyses were the cross-sections with the smallest lumen area. The minimal lumen and vessel diameter, lumen, and internal elastic membrane (IEM) areas were measured. The neointimal area (difference between IEM and lumen area) and percent area stenosis (%AS=neointimal area/IEM area *100) were calculated.

*Supplemental Method, Histopathology and histomorphometry*

Inflammation, hemorrhage, and necrosis scores and endothelialization were identified in each segment by an experienced pathologist, blinded for randomization [1-4]. Fibrin and collagen deposition of the intima, media, and adventitia was calculated using computerized planimetry after picrosirius red staining. The vessel injury score was determined by the anatomic vessel structures similar to the post-stenting injury score [2]. The score was adapted for balloon injury only. For injury score, a numeric value was assigned for each vessel, as follows: Grade 0 (no injury): internal elastic lamina (IEL), external elastic lamina (EEL) and media intact; Grade 0.05: IEL minimal disruption, media and EEL intact; Grade 1: IEL lacerated, media and EEL intact; Grade 1.5: IEL lacerated, media <half thickness lacerated, EEL intact; Grade 2: IEL lacerated, media >half thickness lacerated, EEL intact; Grade 2.5: IEL and media (full thickness) lacerated, EEL minimal disruption; Grade 3: IEL, media (full thickness) and EEL lacerated. Inflammation, hemorrhage, or necrosis scores were calculated as follows: 0 to 3 for non-existent, mild, moderate, or heavy changes, as non-existent, <10%, 10–25%, or >25% of the vessel circumference, respectively. An additional inflammatory score of 4 was given for granulomatous inflammatory reaction in any layer of the artery. Endothelialization was determined with a score system including absent, partial, or complete.

Quantitative histomorphometric parameters included (1) lumen area, (2) IEL area, (3) EEL area, and (4) maximal neointimal thickness of the dilated segment and proximal and distal reference segments. Calculated histomorphometric parameters included (1) neointima area (difference between IEL and lumen area), (2) media area (difference between EEL and IEL area), and (3) % area stenosis [(neointimal area/IEL area)*100].

To investigate the expression of the proinflammatory cytokine tumor necrosis factor alpha (TNF-alpha) in the acute injured vessel, immunofluorescence staining of the cross-sections of the vessel at the highest injury site 1 h after balloon dilation was performed using anti-TNF-alpha antibody (ABCAM Cambridge, UK) and the secondary antibody of goat anti-rabbit Alexa Fluor 594 (Abcam Cambridge, UK). Alpha actin (ABCAM Cambridge, UK) staining of the smooth muscle cells was performed 32±2 days after balloon dilation. For nuclear counterstaining, DAPI (Thermo Fischer Scientific Inc. Rockford, IL, USA) was used. Fluorescent images were acquired by an Olympus Provis AX 70 microscope (New Hyde Park, NY).

***Supplemental Limitations***

We are aware that the time of the balloon inflation might be longer than 2 min in the peripheral arteries; however, all preclinical studies with other types of DCB used 30 to 60 s of balloon inflation time. To compare the drug uptake in the vessel wall with other DCB studies, we also used a 60-s inflation time, but the vascular response and remodeling studies were performed with 2-min inflation time, which is more similar to human conditions.

OCT is suited for phenotypic near-field intravascular imaging and for understanding the vascular response to implanted devices or intravascular therapies. OCT was useful for measuring actual lumen dimensions in vivo and was more accurate than angiography. OCT has the fundamental limitation that it cannot accurately measure the atherosclerotic lesion across the vessel wall because of the limited penetration of the OCT light through the plaque of heavily diseased segments. Furthermore, the external elastic membrane border cannot be delineated in large vessels and severe plaques. However, our study included no calcified or severe diseased vessels, as confirmed by histology.

In contrast with the parallel existence of both adaptive and constrictive remodeling of the long human atherosclerotic femoral arteries, the femoral vessels of the pigs are much shorter; therefore, the most narrowed segment could easily be localized by quantitative angiography and OCT.

Previously published preclinical and clinical data regarding DCB use in peripheral arteries have shown results that are complementary to those of our present study. These studies have differed in design, interventional procedures, drug tested, and species investigated, with each providing unique data that are useful for clinical practice. Data from clinical studies using DCB in peripheral arteries show favorable endpoint outcomes, such as a high grade of primary patency; low incidence of major adverse events (the composite of death, amputation, or revascularization); and positive changes in ankle–brachial index, Rutherford class, claudication distance, and quality of life, demonstrating an undoubted clinical value of this technique. Considering the different study endpoints, a preclinical and a clinical study a priori cannot report similar endpoint results. Preclinical studies, such as our present investigation, aim to provide greater insight into pathological and pathophysiological processes and are necessary for evaluation of the safety of intravascular devices.

***Supplemental References***

1. Rosenthal EA, Bohlmeyer TJ, Monnet E, MacPhail C, Robertson AD, Horwitz MA, Burchenal JE, Horwitz LD (2001) An iron-binding exochelin prevents restenosis due to coronary artery balloon injury in a porcine model Circulation 104:2222-2227.
2. Schwartz RS, Edelman E, Virmani R, Carter A, Granada JF, Kaluza GL, Chronos NA, Robinson KA, Waksman R, Weinberger J, Wilson GJ, Wilensky RL (2008) Drug-eluting stents in preclinical studies: updated consensus recommendations for preclinical evaluation. Circ Cardiovasc Interv 1:143-153. doi: 10.1161/CIRCINTERVENTIONS.108.789974.
3. Thim T, Hagensen MK, Drouet L, Bal Dit Sollier C, Bonneau M, Granada JF, Nielsen LB, Paaske WP, Bøtker HE, Falk E (2010) Familial hypercholesterolaemic downsized pig with human-like coronary atherosclerosis: a model for preclinical studies. EuroIntervention 6:261–268. doi: 10.4244/.
4. Virmani R, Farb A (1999) Pathology of in-stent restenosis. Curr Opin Lipidol 10:499-506.
